# Involved Site Radiotherapy Extends Time to Premature Menopause in Infra-Diaphragmatic Female Hodgkin Lymphoma Patients – An Analysis of GHSG HD14- and HD17-Patients

**DOI:** 10.3389/fonc.2021.658358

**Published:** 2021-05-25

**Authors:** Johannes Rosenbrock, Andrés Vásquez-Torres, Horst Mueller, Karolin Behringer, Matthias Zerth, Eren Celik, Jiaqi Fan, Maike Trommer, Philipp Linde, Michael Fuchs, Peter Borchmann, Andreas Engert, Simone Marnitz, Christian Baues

**Affiliations:** ^1^ Department of Radiation Oncology, CyberKnife and Radiation Therapy, Faculty of Medicine and University Hospital Cologne, University of Cologne, Cologne, Germany; ^2^ Radiation Therapy Reference Center of the German Hodgkin Study Group (GHSG), Faculty of Medicine and University Hospital Cologne, University of Cologne, Cologne, Germany; ^3^ German Hodgkin Study Group, Faculty of Medicine and University Hospital Cologne, University of Cologne, Cologne, Germany; ^4^ Department of Hematology and Oncology, Faculty of Medicine and University Hospital Cologne, University of Cologne, Cologne, Germany

**Keywords:** Hodgkin lymphoma, involved site radiotherapy, involved field radiotherapy, chemotherapy, fertility, premature menopause, infradiaphragmatic

## Abstract

**Introduction:**

Consolidation radiotherapy in intermediate stage Hodgkin´s lymphoma (HL) has been the standard of care for many years as involved field radiotherapy (IFRT) after chemotherapy. It included initially involved region(s). Based on randomized studies, radiation volumes could be reduced and involved site radiation therapy (ISRT) became the new standard. ISRT includes the initially affected lymph nodes. In young adults suffering from HL, infertility and hypogonadism are major concerns. With regard to these questions, we analyzed the influence of modern radiotherapy concepts such as consolidating ISRT in infradiaphragmatic involvement of HL after polychemotherapy.

**Patients and Methods:**

Five hundred twelve patients treated within German Hodgkin Study Group (GHSG) HD14 and HD17 trials were evaluated. We analyzed log-adjusted follicle-stimulating-hormone (FSH)- and luteinizing-hormone (LH)-levels of HD14-patients with infradiaphragmatic radiotherapy (IDRT) in comparison with HD14-patients, who had a supradiaphragmatic radiotherapy (SDRT). In a second step, we compared IFRT with ISRT of female HD17 patients regarding the effects on ovarian function and premature menopause.

**Results:**

We analyzed FSH- and LH-levels of 258 female and 241 male patients, all treated with IFRT. Of these 499 patients, 478 patients had SDRT and 21 patients had IDRT. In a multiple regression model, we could show that log-adjusted FSH (p=0.0006) and LH values (p=0.0127) were significantly higher after IDRT than after SDRT. The effect of IDRT on gonadal function was comparable to two cycles of escalated bleomycin, etoposide, doxorubicin, cyclophosphamide, vincristine, procarbazine, and prednisone (BEACOPPesc). We compared the effect of IFRT with ISRT in thirteen female HD17 patients with infradiaphragmatic (ID) involvement. The mean ovarian dose after ISRT was significantly lower than after IFRT. The calculated proportion of surviving non-growing follicles (NGFs) increased significantly from 11.87% to 24.48% in ISRT compared to IFRT, resulting in a significantly longer calculated time to menopause. The younger the age at therapy, the greater the absolute time gain until menopause.

**Conclusion:**

Infradiaphragmatic IFRT impairs gonadal function to a similar extent as two cycles of BEACOPPesc. In comparison, the use of ISRT target volume definition significantly reduced radiation dose to the ovaries and significantly extends the time interval from treatment to premature menopause.

## Introduction

Nowadays Hodgkin´s Lymphoma (HL) is a very well curable disease. Due to improved overall survival of more than 90% after 5 years for early stage HL (1–4) and even advanced stages (5, 6), reduction of long-term side effects became more and more important. Therefore, recent studies focused on de-escalation of both treatment modalities- radiotherapy (RT) and chemotherapy (1, 3). In the young adult population suffering from HL, infertility and hypogonadism are major concerns, which affect quality of life as well as life planning, desire to have children, and parenthood. Several studies have investigated the effects of chemotherapy and RT on infertility and hypogonadism in HL patients. However, whereas there are studies on current chemotherapy regimens (7–9), the studies on RT date back to the extended field radiotherapy (EFRT) era (10–13).

For the last two decades, consolidating RT in intermediate stage HL was performed as involved field radiotherapy (IFRT) and no longer as EFRT as part of the combined modality treatment (14). It included one or more initially involved regions after completion of polychemotherapy with two cycles of escalated bleomycin, etoposide, doxorubicin, cyclophosphamide, vincristine, procarbazine, and prednisone (BEACOPPesc) and two cycles of doxorubicin, vinblastine, dacarbazine, and bleomycin (ABVD) (2). Based on the ILROG (international Lymphoma Radiation Oncology Group) guidelines for target volume definition in HL involved site radiotherapy (ISRT) has been established since 2014 as new standard (4, 15, 16). Instead of irradiating the initially affected areas, only the initially affected lymph nodes with a margin dependent on the uncertainty in contouring are included in the ISRT target volume (15), which reduces the planning target volume by about half - at least in the case of supradiaphragmatic radiotherapy (SDRT) (17).

The observation of cancer childhood survivors showed that the testis (18) and the ovaries are very sensitive to RT (19–21). The high radiation sensitivity of the gonads has also been detected in adult cancer survivors (10–13, 22, 23). In case of irradiation of the ovaries, a certain percentage of follicles will survive depending on the applied dose, so that the time until the onset of ovarian insufficiency depends not only on the applied dose but also on the pre-existing ovarian reserve of the patient (24). Wallace et al. developed a model to calculate the survival fraction of non-growing follicles (NGF) as a function of dose. He showed that the median lethal dose (LD50), which destroys 50% of the NGF, is < 2Gy (25). Ovarian failure causes not only infertility but also premature menopause. This in turn can lead to increased cardiovascular risk (26, 27), osteoporosis (28) with a risk of bone fracture (29), and reduced quality of life (30).

In this study, we investigate the effects of radiotherapy on Infertility and Hypogonadisms in HL patients and the chance of reducing this impact by using ISRT.

## Patients and Methods

**Figure 1:**
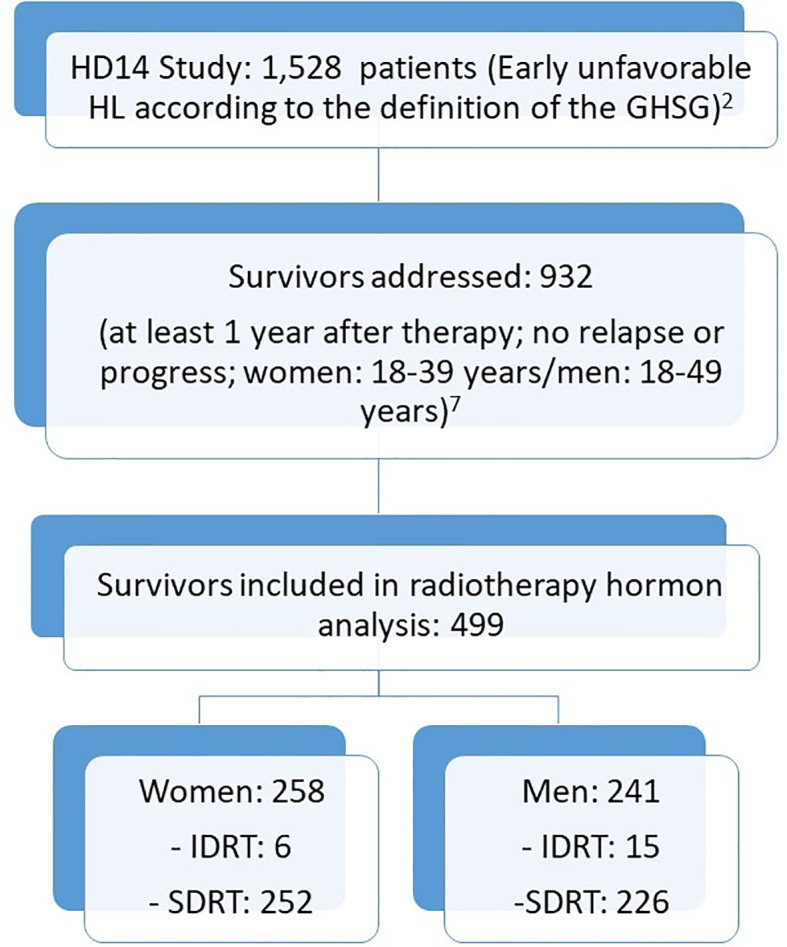
Consort Diagram: hormone analysis.

To evaluate the effects of infradiaphragmatic involved-field radiotherapy (IDRT), we analyzed hormone levels of German Hodgkin Study Group (GHSG) HD14-patients (**Figure 1**). In a second step, we compared IFRT and ISRT of female HD17-patients with regard to premature menopause.

### Hormone Levels

In the HD-14 trial 1,528 patients with early unfavorable HL were included. The treatment consisted of either 4xABVD or 2xBEACOPPesc + 2xABVD (2 + 2-regime) followed by 30 Gy IFRT (2). Behringer et al. determined FSH, LH and AMH/Inhibin B-values of 1,323 patients, who had participated in the HD13-15-trials and had finished chemotherapy at least one year before. They included female patients who were younger than 40 years at the time of diagnosis and male patients who were younger than 50 years at the time of diagnosis but were still in remission and receiving no therapy other than the study medication (7).

To evaluate the effects of RT on gonadal function we compared FSH and LH values of HD-14 patients from this collective who received IDRT with the values of the patients radiated with SDRT. Patients from whom the information on irradiation, FSH and LH levels were available were considered.

### Hormone Levels - Statistics

To achieve a normalized distribution of FSH and LH we took the natural logarithm of the hormone levels. We analyzed the effects of IDRT versus SDRT on log-transformed FSH value. For the analysis, we used a multiple regression model with adjustment for age, gender and chemotherapy (ABVD versus 2 + 2-regime) and computed best linear unbiased estimates to account additionally for the interaction between age and gender. We set the level of significance to 0.05, computed two-sided p values and used SAS 9.4 for the statistical analysis of hormones.

### Comparison IFRT and ISRT

In the subsequent HD17-trial for early unfavorable HL, patients receive 2xBEACOPPesc + 2xABVD. After a post-chemotherapy PET-CT, patients were treated with IFRT in the standard am regardless of the PET-CT result. In the experimental arm, INRT was performed if the patient was PET-CT positive. If the PET-CT was negative, radiotherapy was omitted.

For our analysis, we chose female patients, who had provided us with both initial CT-Scans, pre-radiotherapy-CT-Scans, and who had no oophorectomy. For each patient we contoured an IFRT Planning Target Volume (PTV) in accordance with the definition of the GHSG and an ISRT-PTV according to the ILROG. We chose ISRT and not INRT due to the higher clinical relevance after the implementation of ISRT in several guidelines (15). We calculated for both PTVs a Volumetric Arc Therapy (VMAT)- plan with VARIAN Eclipse 13.6 and analyzed ovaries, uterus, small bowl, rectum, bladder, spinal cord and femoral heads as organs at risk (OAR).

### Comparison IFRT and ISRT – Influence on Premature Menopause

Using Wallace’s surviving percentage function (24) *log*_10_ (*g*(*z*)) = 2–0.15z, we calculated for each patient the percentage of NGF surviving the IFRT and ISRT (g = surviving NGF in %; z dose in Gray). For this, the dose-volume histograms (DVHs) of both ovaries together were analyzed with an interval width of 0.1 Gray and Wallace’s survival function was applied to the resulting data:

g=∑i=1dmax(102−0.15∗(12∗(di+di+1))∗vivboth ovaries)

Based on a theoretical age at the beginning of therapy of 18 to 48 years in two-year steps, we calculated the expected time to menopause for each age and each resulting percentage of NGF. For this, we used Hansen’s model (31) *log*_10_ (*n*) = (–0.00019)*(*age in years*)^2.452^ + 5.717 to calculate an initial value for NGF for each age between 18 and 48 years. For each patient, we multiplied this baseline with the calculated percentage of surviving follicles after IFRT and ISRT to calculate the reproductive age using the Hansen model. According to Wallace (24), the time of menopause was determined by subtracting reproductive age of 50.4 years.

### Comparison IRT and ISRT - Statistics

We compared the mean dose of the OARs, the mean of surviving NGFs and the mean of the time to menopause. Since the mean values of the examined parameters were not normally distributed and not symmetrically distributed, we used a sign test.

## Results

### Hormone Levels

We analyzed FSH and LH levels of 258 female and 241 male HD-14 patients. Of 258 female patients, six women were treated with IDRT and 252 women with SDRT. Of 241 male patients, 15 men had been radiated with IDRT and 226 men with SDRT (**Table 1**).

**Table 1:** Frequency of IDRT/SDRT.

	Female		Male		Total	
	N	%	N	%	N	%
IDRT	6	2.3	15	6.2	21	4.2
SDRT	252	97.7	226	93.8	478	95.8
Total	258	100.0	241	100.0	499	100.0

Hormone levels were taken in mean at 41.4 months (Standard deviation 19.2 months) after RT was performed. **Tables 2A** and **2B** show FSH and LH values stratified by irradiation type and age.

**Table 2a:** Hormone statistics in HD14 according to age group; Standard deviation (Std Dev).

	Original units, U/l				Log Transformed			
	Age < 30 years		Age ≥ 30 years		Age < 30 years		Age ≥ 30 years	
	Mean	Std Dev	Mean	Std Dev	Mean	Std Dev	Mean	Std Dev
FSH fem	6.0	4.6	16.1	23.7	1.3	1.2	1.9	1.5
FSH male	7.1	8.0	8.7	8.1	1.6	0.8	1.9	0.8
LH fem	6.0	7.9	11.1	13.5	1.0	1.6	1.6	1.6
LH male	5.3	3.0	5.4	3.5	1.5	0.5	1.5	0.6

**Table 2b:** Hormone statistics in HD14 according to SDRT *vs*. IDRT; Standard deviation (Std Dev).

	Original units, U/l				Log Transformed			
	SDRT		IDRT		SDRT		IDRT	
	Mean	Std Dev	Mean	Std Dev	Mean	Std Dev	Mean	Std Dev
FSH fem	11.9	19.3	17.3	10.0	1.7	1.4	2.5	1.1
FSH male	7.9	8.0	14.3	8.6	1.7	0.8	2.4	0.7
LH fem	8.9	11.9	11.9	8.4	1.4	1.6	2.2	0.9
LH male	5.2	3.4	7.1	3.2	1.5	0.6	1.9	0.5

Using a multiple regression model we found that the adjusted log values of FSH (FSH p= 0.0006) and LH (p= 0.0127) were significantly higher after IDRT than after SDRT. Apart from radiotherapy, type of chemotherapy, age and interaction of age and gender influenced hormone levels significantly (**Table 3**).

**Table 3:** Multiple regression model of log. FSH and log. LH (weighted least squares, 258 females and 241 males, best linear unbiased estimators).

	Log FSH		Log LH	
	regression coefficient	p-value	regression coefficient	p-value
Intercept	2.069	<.0001	1.624	<.0001
Age(z)	0.039	<.0001	0.033	<.0001
Sex(z)	0.101	0.0816	-0.036	0.7455
Age*Sex(z)	-0.040	0.0027	-0.056	0.0002
ABVD (vs. 2 + 2)	-0.634	<.0001	-0.243	0.0002
Infra- diaphragmatic RT fields (vs. upper fields)	0.598	0.0006	0.349	0.0127

**Figure 2:**
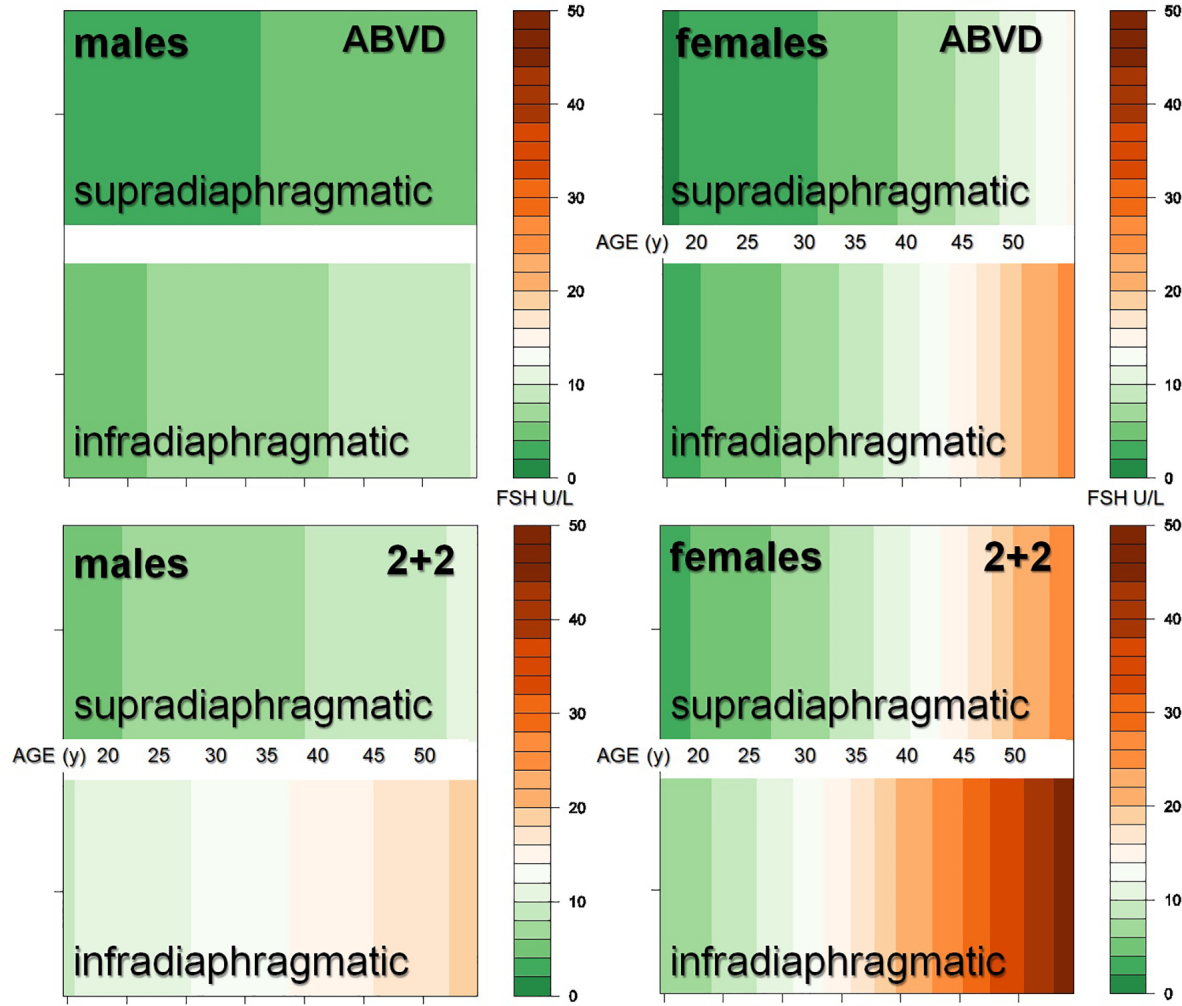
FSH by RT-Field, chemotherapy, gender, and age as estimated in the multiple regression model of **Table 3**.

As the consequences of the multiple regression model in **Table 3** and especially the observed interaction effect are difficult to understand, we used the R package visreg (https://cran.r-project.org/web/packages/visreg/visreg.pdf) to visualize the consequences of the model for FSH levels (**Figure 2**). As shown in **Figure 2**, increased FSH values were present after IDRT and especially women were negatively affected. As the effects of infradiaphragmatic RT versus supradiaphragmatic RT and 2 + 2 versus ABVD were statistically coded in the same way, the similar regression coefficients (0.634 and 0.598) in **Table 3** correspond to comparable effect sizes. The combination of infradiaphragmatic RT and 2 + 2 causes significantly increased FSH values even in younger women. At the other side, males and their FSH levels are clearly less affected, even if heavily treated with 2 + 2, IDRT and in higher age.

### Comparison IFRT and ISRT

The imaging required for analysis was available for 13 HD17-patients, so that we were able to include these patients in the plan comparison. The initial involvement of the patients is shown in **Table 4**. We could show that in comparison with IFRT the use of ISRT significantly reduced the mean ovarian dose from 15.13 Gy to 7.44 Gy (**Table 5**). The mean dose exposure in uterus decreased significantly from 14.51 Gy to 8.94 Gy. The mean dose in all other risk organs studied was also significantly lower with ISRT than with IFRT.

**Table 4:** Initial nodal involvement.

Nodal involvement	N	%
Iliacal right	4	30.8
Inguinal right	4	30.8
Iliacal left	11	84.6
Inguinal left	10	76.9
paraaortic	9	69.2
Celiac	2	15.4
Mesenteric	4	30.8
Liver	0	0.0
Hepatic hilum	1	7.7
Spleen	0	0.0
Splenic hilum	0	0.0

**Table 5:** Organs at risk.

	Involved Field		Involved Site		p-value
	Mean	Standard deviation	Mean	Standard deviation	
Small bowel (D_mean_ in Gy)	13,29	1,52	7,88	2,65	<0.001
Bladder (D_mean_ in Gy)	11,67	3,56	8,74	3,36	0.003
Femoral head left (D_mean_ in Gy)	17,51	4,10	14,47	4,89	0.022
Femoral head right (D_mean_ in Gy)	9,40	7,23	5,02	5,13	<0.001
Rectum (D_mean_ in Gy)	10,03	4,01	6,44	3,48	<0.001
Spinal cord (D_max_ in Gy)	18.88	1.43	15.06	2.85	<0.001
Uterus (D_mean_ in Gy)	14,51	5,41	8,94	4,43	<0.001
Ovaries (D_mean_ in Gy)	15,13	6,34	7,44	5,64	<0.001
Surviving NGF (in %)	11,87	13,01	24,48	12,69	<0.001

**Figure 3:**
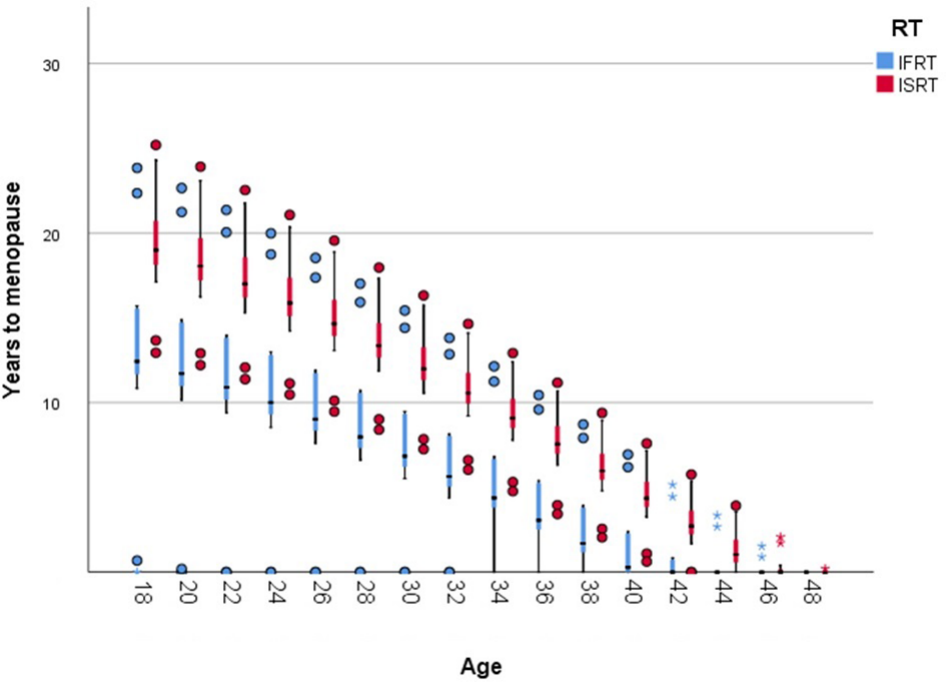
Time to menopause by age and RT-Field; Boxplot (minimum, first quartile, median, third quartile, and maximum; Dots represent outliers greater or less than the 1.5 x interquartile range).

The proportion of surviving NGF increased significantly from 11,87% after IFRT to 24,48% using ISRT. Up to an age of 44 years, the calculated premature menopause occurs significantly later after ISRT than after IFRT (**Table 6** and **Figure 3**). After 44 years this difference is no longer significant.

**Table 6:** Time to menopause.

Age, years	Involved Field Radiotherapy – time to menopause		Involved Site Radiotherapy – time to menopause		Time-Difference of Involved Field and Involved Site radiotherapy		p-value
	Mean, years	Standard deviation, years	Mean, years	Standard deviation, years	Mean, years	Standard deviation, years	
18	12.86	6.82	19.16	3.49	6.30	5.35	<0.001
20	12.13	6.56	18.19	3.33	6.06	5.18	<0.001
22	11.34	6.21	17.13	3.18	5.79	4.91	<0.001
24	10.49	5.82	15.97	3.03	5.48	4.57	<0.001
26	9.57	5.40	14.73	2.88	5.16	4.21	<0.001
28	8.59	4.96	13.42	2.73	4.83	3.84	<0.001
30	7.55	4.51	12.04	2.59	4.49	3.44	<0.001
32	6.45	4.06	10.60	2.46	4.15	3.05	<0.001
34	5.31	3.61	9.11	2.33	3.80	2.66	<0.001
36	4.12	3.18	7.57	2.21	3.45	2.30	<0.001
38	2.89	2.79	5.99	2.10	3.10	2.00	<0.001
40	1.72	2.38	4.37	2.00	2.65	1.68	<0.001
42	0.95	1.75	2.81	1.71	1.87	1.23	0.001
44	0.46	1.14	1.37	1.27	0.90	0.71	0.001
46	0.19	0.47	0.33	0.71	0.15	0.27	0.125
48	0.00	0.00	0.02	0.06	0.02	0.06	1.000

## Discussion

By intensifying chemotherapy over the years, the HL specific mortality decreased. Current therapeutic strategies lead to 5-years OS-rates above 90-95% for early-stage favorable and unfavorable HL (1–4). Considering this, the toxicity of the combined modality treatment plays a crucial role and researcher are trying to reducing these long-term effects. An important step in the reduction of side effects in the context of radiotherapy, was the change from EFRT to IFRT and finally to ISRT. This gradual reduction of the RT fields led to a consecutive reduction of the irradiated volume and a lower exposure of the organs at risk. Especially in young HL-patients, hypogonadism and infertility are particularly important issues, which can be caused by chemotherapy or radiotherapy. The use of ISRT could help to reduce this particularly relevant toxicity.

We could show that log-adjusted FSH and LH values were significantly higher after infradiaphragmatic IFRT than after supradiaphragmatic IFRT. The negative effect of infradiaphragmatic IFRT on gonadal function was comparable to the effect of two cycles of BEACOPPesc. Our comparison between IFRT and ISRT indicated that the mean ovarian dose was significantly lower and the calculated time to menopause was significantly longer after infradiaphragmatic ISRT than after infradiaphragmatic IFRT. The younger the age at therapy, the greater the absolute time gain until menopause.

Our evaluation of the hormone levels of infradiaphragmatic IFRT treated HD14 patients confirms that women in particular have a high risk of premature onset of hypogonadism and the effect is comparable to the effect of 2 cycles of BEACOPPesc. These results correspond with the outcome of other groups. Van der Kaaij analyzed 460 female HL-survivors. Forty-one percent (11/27) of the patients treated with iliacal RT suffered from premature ovarian failure. However, all of them had also been treated with alkylating chemotherapy (10). De Bruin et al. calculated the cumulative risk for menopause at the age of 40 years by examining a collective of 549 women after HL-Therapy. Thirty-one women had been treated with RT only, which included the ovaries. Thirteen of these 31 patients developed a menopause before they had reached the age of 40 years (12). Moreover, our results fit well with the model designed by Wallace, in which the ovarian reserve after RT depends on two independent factors: dose and age (24).

A weakness of our analysis is the small number of patients who were irradiated infradiaphragmatic. On the other hand, the studies described in the literature are also based on very few patients, so that they were not statistically analyzed (10–13). Since we examined a well-defined collective of a clinical study, we were able to demonstrate a highly significant difference independent of the chemotherapy used, despite the small number of patients. Furthermore, our study is - to our knowledge - the first to investigate the effect of infradiaphragmatic IFRT on gonodal function in post EFRT era.

Due to the proven high gonadal toxicity of infradiaphragmatic IFRT, it is important to find ways to reduce it. Planning studies show that with supradiaphragmatic INRT/ISRT (17, 32, 33) and infradiaphragmatic ISRT (33) second malignancy risk is significantly lower than with IFRT. To date, however, no studies have investigated the effect of infradiaphragmatic ISRT/INRT on fertility. Knowing the high gonadal toxicity of infradiaphragmatic IFRT in women, the evaluation of ISRT is of high importance.

To our knowledge, our study is the first, which investigated the opportunity of reducing gonadal toxicity by using ISRT for infradiaphragmatic HL involvement. Our results show that the mean dose in the ovaries is significantly lower with ISRT than with IFRT and furthermore, the predicted percentage of surviving NGFs is significantly higher after ISRT. This is reflected in the clinically relevant time to premature menopause after RT. Using Wallace’s survival model for NGFs (24) and Hansen’s NGF model for age (31) we could demonstrate that the time to menopause is significantly longer after ISRT than after IFRT. This is particularly evident in younger women.

A limitation of our analysis is that we performed a plan comparison in only 13 patients. However, small numbers of patients are common in planning studies. Furthermore, we were able to demonstrate an advantage for ISRT in every single patient. A hormonal analysis of patients with IFRT versus ISRT in a prospective study would certainly be preferable. However, since ISRT has meanwhile replaced IFRT as the standard, this would be difficult to realize.

A further weakness of our analyzed patient cohort was that patients had not received ovarian transposition prior to radiotherapy. Some studies have shown that oophoropexy can preserve ovarian function despite large-volume IDRT (34–36). However, in other publications, premature menopause could not be prevented by ovarian transposition (37, 38). One reason for this was possibly the scattered radiation, which in the case of paraaortic RT probably resulted in a relevant dose exposure to the ovaries despite oophoropexy. This is especially true for modern irradiation techniques such as VMAT, as the irradiation is not strictly appa oriented. This results in optimized dose coverage of the target volume, but at the cost of some low-dose exposure in the area of the organs at risk, and in this case, the ovaries. Therefore, the combination of ISRT and oophoropexy may lead to the necessary reduction of the ovarian dose.

Furthermore, the use of ISRT reduced significantly the mean dose of the uterus. Studies of childhood cancer survivors have reported that radiation of the uterus in childhood can lead to severe dysfunction (39, 40). In adults, pregnancies following pelvic radiation are very rare and only few case of successful pregnancies after RT have been reported (41). Accordingly, a premenopausal irradiated uterus presents on MRI imaging similar to a postmenopausal uterus (42). Overall, it is assumed that irradiation of the uterus can lead to infertility, miscarriages, premature births, and low birth weight even in adult women (43). This is caused by damage to the endometrium, which impedes implantation of the ovum, damage to the uterine vessels and fibrosis of the myometrium, which cause growth retardation and thus early abortion (43). Besides the low ovarian dose, the significant reduction of the uterine dose makes pregnancy more likely after ISRT compared to IFRT. Therefore, we assume that the positive effect of oophoropexy on fertility can be further enhanced by ISRT.

In summary, the use of ISRT prolongs the time to early menopause. Since premature menopause can lead to increased cardiovascular risk (26, 27) and osteoporosis (28, 29), it can be supposed that these effects can be reduced by the use of ISRT. Furthermore, due to the less reduced ovarian reserve and the lower uterine dose, it can be assumed that the probability of pregnancy after infradiaphragmatic ISRT is clinically relevant higher than after infradiaphragmatic IFRT.

Another point to be discussed is the early onset of menopause in many women after chemotherapy. This could be an indication that the sparing of the ovaries during radiation may not seem to be so relevant. However, Behringer et al. reported that compared to 6-8 cycles of BEACOPPesc after the 2 + 2-regime and especially after ABVD, there are fewer limitations of gonadal function (7). In another analysis by Behringer et al, the 2 + 2 regimen and ABVD did not differ in terms of pregnancies, offspring, amenorrhea, and menopausal symptoms. After adjustment for age, there was no difference between the patients analyzed and the general German population in terms of motherhood rates (8). Nevertheless, we could show that especially the combination of the 2 + 2 regimen and infradiaphragmatic IFRT leads to a significant reduction of ovarian reserve even in young women. Therefore, especially in the early favorable and early unfavorable stage, ovarian sparing should be aimed at.

In conclusion, we demonstrated that the long-term effects of infradiaphragmatic IFRT on ovarian function are comparable to the effects of BEACOPP_esc_ in the 2 + 2 regimen and the combination of both causes early menopause even in young women. Therefore, optimal sparing of the ovaries should always be performed in the combined modality treatment. In addition, we have been able to report how large the effect of an optimized target volume definition is. By using ISRT, a significant reduction of the radiotherapy volume can be achieved, which plays a crucial role with regard to the improved sparing of the ovaries and the consecutive ovarian function. It is assumed that in the future immunotherapy will play a greater role in the primary therapy of HL and this will lead to reduced toxicity of systemic therapy while maintaining the prognosis in early and intermediate stages of HL. Therefore, in the future, even more attention should be paid to the protection of the ovaries during consolidative radiotherapy.

## Data Availability Statement

The raw data supporting the conclusions of this article will be made available by the authors, without undue reservation.

## Ethics Statement

The studies involving human participants were reviewed and approved by Ethikkommission Köln. The patients/participants provided their written informed consent to participate in this study.

## Author Contributions

JR and CB conceived and designed the study. JR, AV-T, CB, and MZ performed the data collection. HM and JR performed the statistical analysis. All authors wrote the manuscript. All authors contributed to the article and approved the submitted version.

## Funding

The collection of hormone levels was supported by Deutsche Krebshilfe (Grant No. 109087), the Bundesministerium für Bildung und Forschung, and the Kompetenznetz Maligne Lymphome.

## Conflict of Interest

The authors declare that the research was conducted in the absence of any commercial or financial relationships that could be construed as a potential conflict of interest.
